# LncRNA MAGI2-AS3 is down-regulated in intervertebral disc degeneration and participates in the regulation of FasL expression in nucleus pulposus cells

**DOI:** 10.1186/s12891-020-3086-y

**Published:** 2020-03-06

**Authors:** Shuting Cui, Zizhen Liu, Bin Tang, Zhizhen Wang, Baojian Li

**Affiliations:** Department of Orthopedics, Liaocheng second People’s Hospital, No. 306 Health Street, Linqing City, Shandong Province 252600 People’s Republic of China

**Keywords:** Intervertebral disc degeneration, lncRNA MAGI2-AS3, Fas ligand

## Abstract

**Background:**

It is known that Fas ligand (FasL) is involved in the development of intervertebral disc degeneration (IDD). A recent study reported that lncRNA MAGI2-AS3 up-regulated the expression of FasL to promote breast cancer. Therefore, we investigated the roles that lncRNA MAGI2-AS3 might play in IDD.

**Methods:**

A total of 66 IDD patients (IDD group) and 58 healthy volunteers (Control group) were recruited in this study. Quantitative real-time PCR (qRT-PCR) and western blot were used to investigate gene expression levels. Cell transfections were carried out to analyze gene interactions. The diagnostic value of lncRNA MAGI2-AS3 for IDD was assessed by ROC curve analysis.

**Results:**

The expression levels of plasma lncRNA MAGI2-AS3 were lower in IDD patients compared to that in the control group. Down-regulation of lncRNA MAGI2-AS3 effectively distinguished IDD patients from the control group. The expression levels of plasma lncRNA MAGI2-AS3 were significantly increased after the treatments. Over-expression of lncRNA MAGI2-AS3 inhibited the expression of FasL, while the silencing of lncRNA MAGI2-AS3 promoted the expression of FasL in nucleus pulposus (NP) cells.

**Conclusions:**

Therefore, lncRNA MAGI2-AS3 is down-regulated in IDD and participates in the regulation of FasL expression in nucleus pulposus (NP) cells.

## Background

Intervertebral disc degeneration (IDD) is a common bone disease that causes lower back pain [1]. IDD is generally considered as the first step of degenerative spinal changes [2]. The development and progression of IDD inevitably leads to disc narrowing, the formation of osteophytes and spinal stenosis [3, 4]. Although various risk factors such as age and obesity have been identified for IDD, the molecular mechanisms of the pathogenesis of this disease are still largely unknown [5]. The incidence of IDD is predicted to be significantly increasing in near future due to lifestyle changes [6]. Therefore, understanding the pathogenesis of IDD is urgently needed to improve treatments of this disease.

Fas ligand (FasL), a member of the tumor necrosis factor family, is a type-II transmembrane protein that can induce cell apoptosis [7]. FasL-induced apoptosis also plays essential roles in the development of IDD [8]. In effect, inhibition of FasL provides new insights into the treatment of IDD [9]. In some cases, FasL interacts with long non-coding RNAs (lncRNAs) to achieve its functions [10]. LncRNAs are a group of non-protein coding RNAs with critical functions involved in various human diseases [11]. It has been reported that lncRNA MAGI2-AS3 can up-regulate FasL to participate in the development of breast cancer [12], indicating the potential involvement of lncRNA MAGI2-AS3 in IDD. In this study we showed that lncRNA MAGI2-AS3 was down-regulated in IDD and negatively regulated the expression of FasL in nucleus pulposus (NP) cells.

## Methods

### Patients

A total of 66 patients with IDD (IDD group, 36 males and 30 females, with the age of 42 to 76 years old and 58.6 ± 4.8 years old, respectively) and 58 healthy volunteers (control group, 31 males and 27 females, with the age of 40 to 77 years and 58.0 ± 5.4 years old, respectively), who were admitted by the Liaocheng Second People’s Hospital from January 2016 to October 2017, were included in this study to serve as research subjects. Inclusion criteria of patients are as follows: 1) patients were diagnosed and treated for the first time; 2) complete medical records; 3) patients who completed treatment in Liaocheng Second People’s Hospital; 4) patients were willing to participate with signed consent form. Exclusion criteria of patients are as follows: 1) patients were diagnosed with multiple diseases; 2) patients were treated before admission. The 58 healthy volunteers received systemic physiological examinations in Liaocheng Second People’s Hospital and all physiological parameters were within the normal range. No significant differences in age and gender were found between the patients and the control groups. This study was approved by the Ethics Committee of Liaocheng Second People’s Hospital.

### Specimens and nucleus pulposus (NP) cells

Fasting blood (5 ml) was extracted from each patient and healthy control 1 day after admission and on the day of discharge. Blood samples were used to prepare plasma samples using the conventional method. NP cells were isolated and cultivated according to the methods described by Wang et al. [13]. Cells were collected from passage 3–5 for subsequent experiments.

### qRT-PCR

To detect the expression of lncRNA MAGI2-AS3, total RNAs were extracted using the TRIzol reagent (Thermo Fisher Scientific). Reverse transcription was performed using the AMV Reverse Rranscriptase (Gibco; Thermo Fisher Scientific). The PCR reaction systems were prepared using the PowerUp™ SYBR™ Green Master Mix (Applied Biosystems™). Primers of lncRNA MAGI2-AS3, FasL and the endogenous control GAPDH were synthesized by GenePharma (Shanghai, China). The expression levels of lncRNA MAGI2-AS3 and FasL were normalized to GAPDH and were calculated according to the 2^-ΔΔCq^ method [14]. .

### Vectors and cell transfection

Vectors expressing lncRNA MAGI2-AS3 and empty vectors were designed and constructed by Sangon (Shanghai, China). The Lipofectamine 2000 reagent (Invitrogen, Thermo Fisher Scientific) was used to perform cell transfections with 10 nM vectors. Cells that were treated with Lipofectamine 2000 reagent only was used as control (C). Transfection with empty vectors was used as negative control (NC).

### Western blotting

To detect the protein expression of FasL, total proteins were extracted using the RIPA solution (Thermo Fisher Scientific). Protein samples were denatured and 10% SDS-PAGE electrophoresis was performed. Proteins were then transferred to PVDF membranes. After blocking in 5% non-fat milk for 1 h, incubation with primary antibodies of rabbit anti-human FasL (1:1000; cat. no. ab15285; Abcam) and anti-GAPDH (1,1000; cat. no. ab8245; Abcam) was performed. Membranes were further incubated with IgG-HRP secondary antibody (goat anti-rabbit, cat. no. sc-2004; Santa Cruz Biotechnology, Dallas, TX, USA). Signals were produced using ECL Western Blotting Substrate (Pierce; Thermo Fisher Scientific). Membranes were scanned using the iBright Imaging Systems Thermo Fisher Scientific). Image J v1.48 was used for data analysis.

### Statistical analysis

Three biological replicates were included in each experiment. Data were expressed as mean ± standard deviation (SD) and were processed using the GraphPad Prism 6 software. Comparisons between IDD and the control group were performed by unpaired t-test. Comparisons between pre-treatment and post-treatment levels of plasma lncRNA MAGI2-AS3 were performed by paired t-test. Comparisons among 3 groups were performed by ANOVA (one-way) and Tukey test. Diagnostic value of lncRNA MAGI2-AS3 for IDD was evaluated by receiver operating characteristic (ROC) curve. Differences with *p* < 0.05 were statistically significant.

## Results

### Plasma levels of lncRNA MAGI2-AS3 were altered in IDD patients

**Fig. 1:**
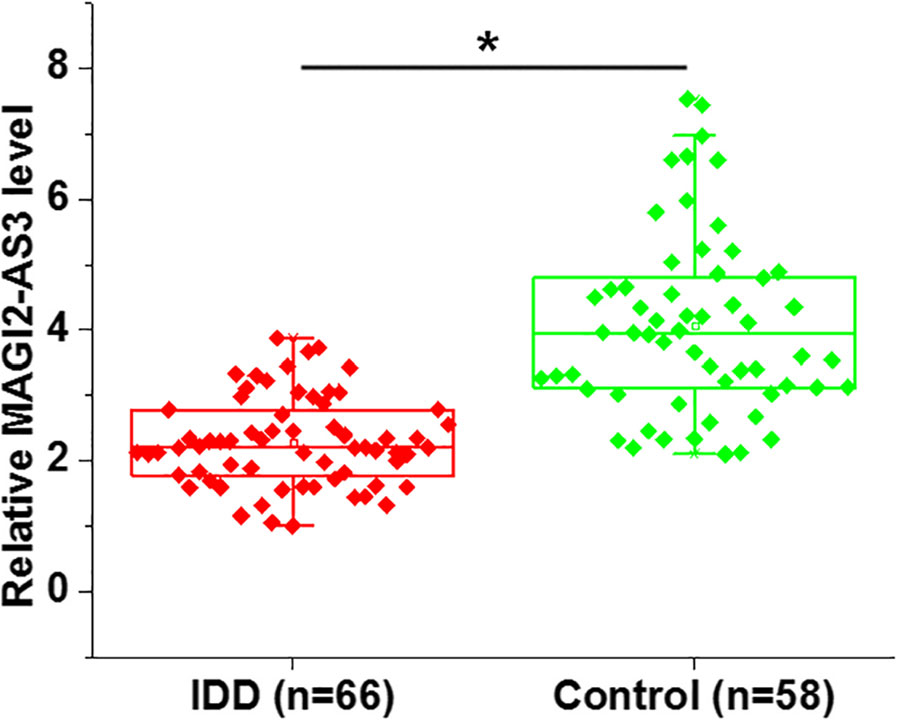
Plasma lncRNA MAGI2-AS3 was significantly down-regulated in IDD patients. qRT-PCR results showed that plasma levels of lncRNA MAGI2-AS3 were significantly higher in IDD patients than that in healthy people (*, *p* < 0.01)

Plasma levels of lncRNA MAGI2-AS were assessed by qRT-PCR in both IDD patients and healthy people. Compared to the control, plasma levels of lncRNA MAGI2-AS3 were significantly lower in IDD patients (Fig. 1, *p* < 0.01).

### Plasma lncRNA MAGI2-AS3 is a potential diagnostic biomarker for IDD

**Fig. 2:**
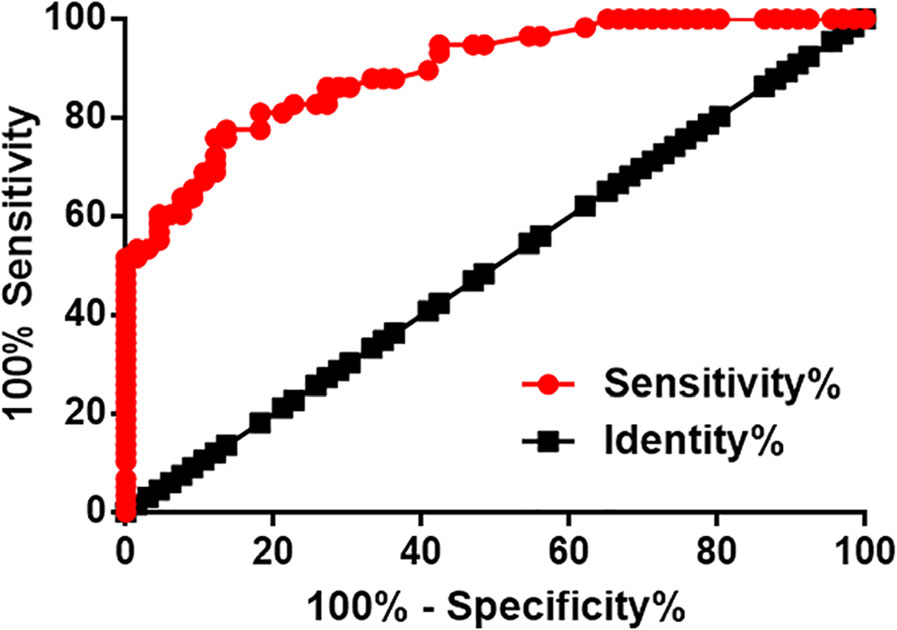
Plasma lncRNA MAGI2-AS3 is a potential diagnostic biomarker for IDD. ROC curve analysis showed that down-regulation of lncRNA MAGI2-AS3 effectively distinguished IDD patients from healthy people

Diagnostic value of lncRNA MAGI2-AS3 for IDD was evaluated by ROC curve analysis with IDD patients as true positive cases and healthy people as true negative cases. The area under the curve was 0.90, with the standard error of 0.027 and 95% confident interval of 0.84–0.95 (Fig. 2).

### Plasma lncRNA MAGI2-AS3 levels were significantly increased after treatment

**Fig. 3:**
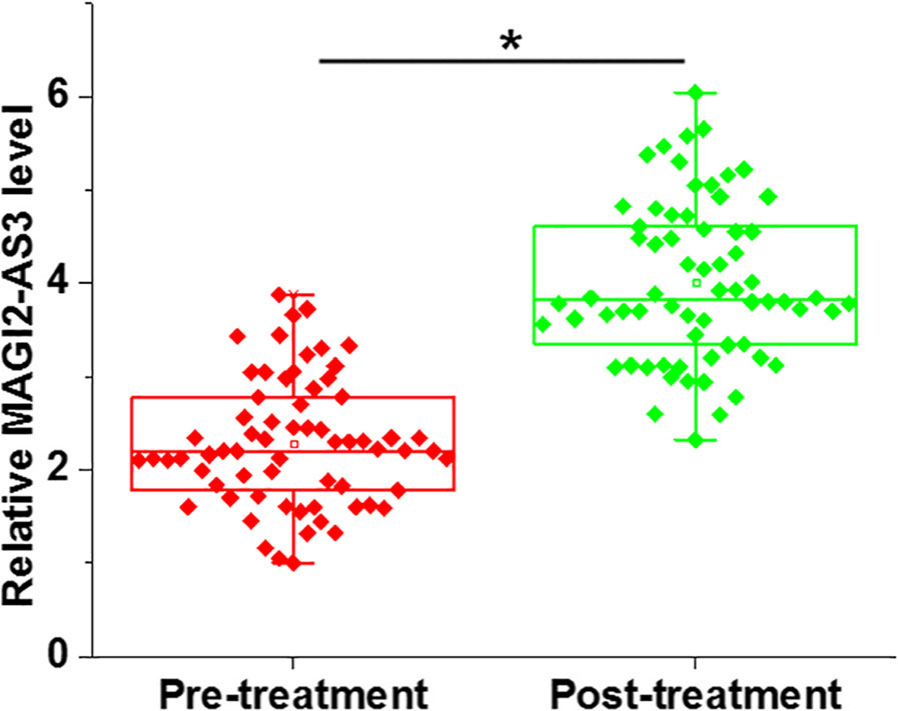
Plasma lncRNA MAGI2-AS3 levels were significantly increased after treatment. qRT-PCR results showed that plasma levels of lncRNA MAGI2-AS3 were significantly increased in IDD patients after treatment compared to that of pre-treatment levels (*, *p* < 0.05)

Results of qRT-PCR showed that the plasma levels of lncRNA MAGI2-AS3 were significantly increased after treatment compared with the pre-treatment levels (*p* < 0.05, Fig. 3,). Therefore, monitoring the levels of plasma circulating lncRNA MAGI2-AS3 may reflect the treatment outcomes.

### LncRNA MAGI2-AS3 negative regulated the expression of FasL in NP cells

**Fig. 4:**
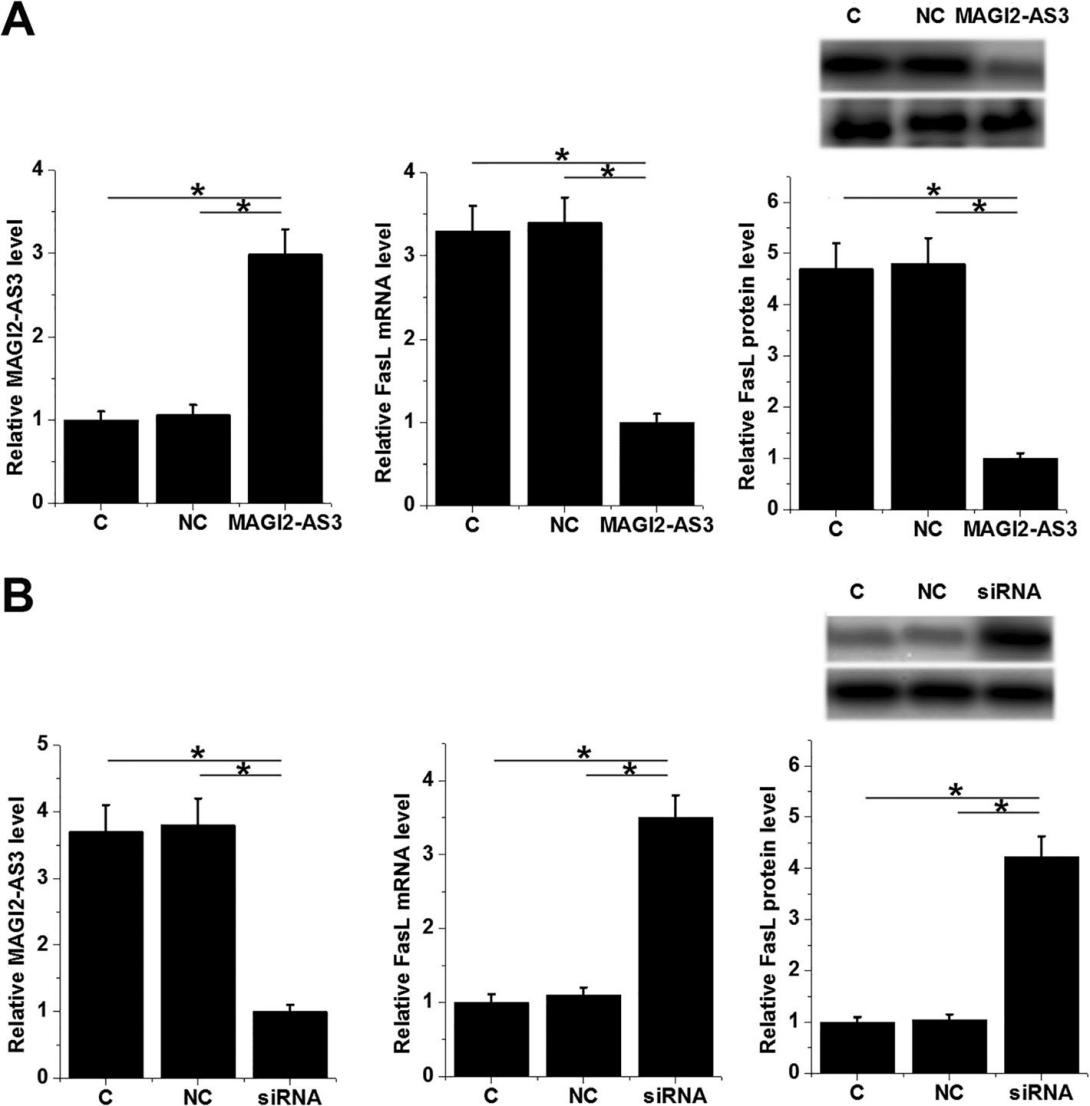
LncRNA MAGI2-AS3 negative regulated the expression of FasL in NP cells. Results of qRT-PCR and western blot showed that over-expression of lncRNA MAGI2-AS3 inhibited (**a**) the expression of FasL, while lncRNA MAGI2-AS3 siRNA silencing promoted (**b**) the expression of FasL in NP cells (*, *p* < 0.05)

Over-expression and knockdown of MAGI2-AS3 were achieved in NP cells, and expression of FasL was detected by qRT-PCR and western blot. Compared to the control (C) and negative control (NC) groups, over-expression of lncRNA MAGI2-AS3 resulted in inhibited expression of FasL in NP cells at both mRNA and protein levels (Fig. 4a, *p* < 0.05). In the contrary, knockdown of lncRNA MAGI2-AS3 played an opposite role (Fig. 4b, *p* < 0.05).

## Discussion

IDD is a common bone disease with unclear pathogenesis. The key findings of the present study are that lncRNA MAGI2-AS3 was down-regulated in IDD, and over-expression of MAGI2-AS3 may improve IDD by down-regulating FasL, which promotes IDD.

The development of IDD is a complex process with multiple internal and external factors involved. Genome-wide transcriptome analysis showed that the development of IDD is characterized by an altered expression pattern of a large set of lncRNAs [15], indicating the involvement of lncRNAs in these diseases [16]. MAGI2-AS3 is a recently identified lncRNA with tumor suppression functions in breast cancer and bladder cancer [12, 17]. In the present study, we showed that plasma circulating MAGI2-AS3 was significantly down-regulated in IDD patients, and down-regulation of MAGI2-AS3 effectively distinguished IDD patient from healthy people. Those findings indicated the involvement of MAGI2-AS3 in IDD, and the potential diagnostic values of circulating MAGI2-AS3 for IDD. However, more clinical trials are needed to further confirm our findings.

NP is the inner core of the vertebral disc, and the elastic inner structure confers vertebral disc the ability to withstand forces of torsion and compression. Accelerated NP cell apoptosis promotes IDD [18]. During the development of IDD, FasL mediates the apoptosis of NP cells, which in turn aggregates disease conditions [13]. In the development of breast cancer, lncRNA MAGI2-AS3 inhibited cancer cell growth by inhibiting FasL [12]. Our results showed that MAGI2-AS3 negatively regulated FasL in NP cells. Therefore, over-expression of MAGI2-AS3 may assist the treatment of IDD by inhibiting FasL.

It is worth noting that in this study we only detected gene expression in plasma, while may not reflect the gene expression status in intervertebral disc tissues. Our future study will try to include intervertebral disc tissue samples to further confirm the dysregulation of MAGI2-AS3 in IDD. In addition, our patients included in this study were all Han Chinese. It is known that ethnicity may also affect gene expression [19]. The roles of MAGI2-AS3 in IDD among different populations should be further explored. Moreover, in vitro experiments of animal model are also expected in future studies to verify the in vivo functions of MAGI2-AS3 in the development of IDD.

## Conclusion

In conclusion, lncRNA MAGI2-AS3 is down-regulated in IDD and participates in the regulation of FasL expression in NP cells.

## Data Availability

The datasets generated and/or analysed during the current study are available in the Baidu Netdisk repository, https://pan.baidu.com/s/1I9XDotFfXyBYravOddKxjg [ACCESSION NUMBER TO DATASETS: a7mw].

## Abbreviations

FasL: Fas ligand
IDD: Intervertebral disc degeneration
lncRNAs: Long non-coding RNAs
NP: Nucleus pulposus
qRT-PCR: Real-time quantitative PCR
